# The Sirt1/P53 Axis in Diabetic Intervertebral Disc Degeneration Pathogenesis and Therapeutics

**DOI:** 10.1155/2019/7959573

**Published:** 2019-09-09

**Authors:** Zengjie Zhang, Jialiang Lin, Majid Nisar, Tingting Chen, Tianzhen Xu, Gang Zheng, Chenggui Wang, Haiming Jin, Jiaoxiang Chen, Weiyang Gao, Naifeng Tian, Xiangyang Wang, Xiaolei Zhang

**Affiliations:** ^1^Department of Orthopaedics, The Second Affiliated Hospital and Yuying Children's Hospital of Wenzhou Medical University, 109 West Xueyuan Road, Wenzhou, 325027 Zhejiang Province, China; ^2^Zhejiang Provincial Key Laboratory of Orthopaedics, Wenzhou, Zhejiang Province, China; ^3^The Second School of Medicine, Wenzhou Medical University, China; ^4^The First Affiliated Hospital of Wenzhou Medical University, Nanbaixiang Street, Ouhai District, Wenzhou, 325027 Zhejiang Province, China; ^5^The Third Affiliated Hospital and Ruian People's Hospital of Wenzhou Medical University, Wansong Road 108#, Ruian, Zhejiang Province, China; ^6^Chinese Orthopaedic Regenerative Medicine Society, Hangzhou, China

## Abstract

Intervertebral disc degeneration (IDD) is one of the major causes of low back pain. Diabetes is a risk factor for IDD and may aggravate IDD in rats; however, the mechanism is poorly understood. Previously, we demonstrated that apoptosis and senescence were increased in diabetic nucleus pulposus (NP) tissues; in the current study, we found that hyperglycaemia may promote the incidence of apoptosis and senescence in NP cells *in vitro*. Meanwhile, the acetylation of P53, a master transcription factor of apoptosis and senescence, was also found increased in diabetic NP tissues in vivo as well as in hyperglycaemic NP cells *in vitro*. Sirt1 is an NAD^+^-dependent deacetylase, and we showed that the expression of Sirt1 was decreased in NP tissues, while hyperglycaemia could suppress the expression and activity of Sirt1 in NP cells. Furthermore, we demonstrated that butein may inhibit acetylation of P53 and protect NP cells against hyperglycaemia-induced apoptosis and senescence through Sirt1 activation, as the Sirt1 inhibitor Ex527 may counteract the protective effect of butein in hyperglycaemic NP cells. An *in vivo* study showed that butein could ameliorate the IDD process in diabetic rats, while Sirt1 was increased and acetyl-p53 was decreased in NP tissues in butein-treated rats. These results indicate that the Sirt1/P53 axis is involved in the pathogenesis of diabetic IDD and may serve as a therapeutic target for diabetic IDD.

## 1. Introduction

Low back pain is one of the most prevalent disorders and the leading cause of disability around the world [1, 2]. Intervertebral disc degeneration (IDD) is widely known to contribute to low back pain [3]. Various factors including aberrant mechanical stress, inflammation, and genetic mutations have been proposed to contribute to the development of IDD [4–7]; however, the cellular and molecular mechanisms of IDD remain unclear, and effective treatments for IDD are still lacking.

The intervertebral disc is an elegant structure composed of the nucleus pulposus (NP), annulus fibrosus (AF), and endplate cartilage (EPC) [8]. Gelatinous NP is the major component of the intervertebral disc, which enables the disc to withstand various mechanical pressures from diverse activities. Additionally, the NP is important for the stabilization and biomechanical maintenance of the discs. Dysfunction of the NP is considered the initiating factor for IDD [9, 10].

Diabetes mellitus is one of the most common metabolic disorders characterized by elevated blood glucose resulting from a deficiency in insulin secretion or insulin resistance [11]. Epidemiological studies have demonstrated that diabetes mellitus is a major risk factor for IDD [12, 13]. A clinical study by Sakellaridis reported that the incidence of surgery for lumber disc disease was significantly higher in diabetic patients than in nondiabetic patients [14]. Recently, an animal study conducted by our group showed that the process of IDD was accelerated in diabetic rats. Moreover, apoptosis and senescence were notably increased in NP tissues from diabetic rats [15]; however, the mechanism through which diabetes induces apoptosis and senescence in NP cells is unclear.

The p53 protein is a transcription factor that regulates approximately 500 target genes. It may control a broad range of cellular processes including cell cycle arrest, DNA repair, and cellular metabolism [16, 17]. Additionally, p53 is a common regulator for senescence and apoptosis [18]. The p53 protein activity is controlled by various forms of posttranslational modifications, including ubiquitylation, phosphorylation, sumoylation, methylation, and neddylation. Furthermore, acetylation is one of the most important modifications necessary for the p53-mediated regulation of cellular senescence and apoptosis [19]. The acetylation of p53 may promote apoptosis and senescence in cells by modulating the expression of Bcl-2, Bax, and p21WAF1, which are crucial proteins involved in apoptosis and senescence [20].

The acetylation modification of p53 is regulated by acetyltransferases and deacetylases. Acetyltransferases such as p300/CBP, PCAF, and MOZ may acetylate p53 at different sites of the C-terminal lysine, while Tip60, MOF, and MOZ may acetylate p53 at lysine 120 (K120), which resides within the DNA-binding domain [19, 21]. Deacetylation of p53 is mainly regulated by histone deacetylase (HDAC) complexes containing HDAC1 and silent information regulator Sirt1 [22]. Sirt1 is an NAD^+^-dependent deacetylase [23], a member of the sirtuin family of proteins and a homologue of the yeast Sir2 protein. Sirt1 has been reported to be associated with many pathophysiological processes, particularly in age-related diseases, such as neurodegenerative disorders and osteoarthritis [24, 25]. The expression of Sirt1 was decreased in degenerated NP tissues in humans as well as in rats [26, 27]. In addition, a substantial amount of evidence has shown that Sirt1 modulates the expression of target proteins and genes involved in the process against senescence and apoptosis [28–30]. Sirt1 can deacetylate many types of key transcription factors and cofactors, such as p53, forkhead box class O (FOXO) proteins, peroxisome proliferator-activated receptor-coactivators (PGC-1), and nuclear factor kappa B (NF-*κ*B), all of which participate in cell cycle arrest and senescence. These factors may affect cellular pathways involved in metabolic diseases such as diabetes [31], indicating that Sirt1 is related to the process of diabetes or diabetes-related diseases. Therefore, we propose that the Sirt1/p53 axis may be involved in the pathogenesis of diabetic IDD and may also serve as a therapeutic target for diabetic IDD.

Hyperglycaemia is an important characteristic of diabetes; herein, we employed hyperglycaemia in our study to investigate the effects of diabetes on NP cells *in vitro*. Streptozotocin- (STZ-) induced diabetic rats were used to study the effects of diabetes on IDD *in vivo*. In this study, we investigated the expression and activity of Sirt1 and the acetylation of p53 in diabetic IDD *in vitro* and *in vivo*. Additionally, we applied butein [32] as a Sirt1 agonist to study its therapeutic potential for diabetic IDD *in vitro* and *in vivo*.

## 2. Materials and Methods

All procedures involving animals were performed under the approval and guidance of the Animal Care and Use Committee of Wenzhou Medical University.

### 2.1. Reagents and Antibodies

Butein (2′,3,4,4′-tetrahydroxy chalcone) was purchased from MedChemExpress (USA). The purity of the compound was ≥99% (HPLC). Streptozotocin (STZ) and citrate buffer and the type II collagenases were from Sigma-Aldrich (St. Louis, MO, USA). A glucometer was purchased from Yuwell Co., China. The primary antibodies of p16, p21, and *β*-actin were acquired from Abcam (Cambridge, UK). The Sirt1, ace-p53, p53, cleaved caspase-3, Bax, and Bcl-2 antibodies were obtained from CST (MA, USA). Alexa Fluor® 488-labeled and Alexa Fluor® 594-labeled Goat Anti-Rabbit IgG (H+L) secondary antibodies were purchased from Jackson ImmunoResearch (West Grove, PA, USA). The 4′,6-diamidino-2-phenylindole (DAPI) was obtained from Beyotime (Shanghai, China). The cell culture reagents were purchased from Gibco (Grand Island, NY, USA).

### 2.2. Sprague-Dawley Rat Model of Diabetes and IDD

Forty-eight adult male SD rats (200–250 g) used for the present study were obtained from the Experimental Animal Institute of Wenzhou Medical University. The rats were housed in a controlled environment under standard conditions of temperature and humidity and an alternating 12 h light and dark cycle. The 48 male SD rats were randomly divided into four groups, namely, the control group (12 males), IDD group (12 males), diabetes IDD group (12 males), and butein-treated diabetes IDD group.

The rat model of diabetes was induced by intraperitoneal injection of STZ (Sigma-Aldrich Co., St. Louis, MO, USA) at 65 mg/kg body weight in 0.1 mol/l citrate buffer (Sigma-Aldrich Co., St. Louis, MO, USA), while the rats in the control group were injected with citrate buffer. Blood glucose level was examined by a glucometer (Yuwell Co., China) on days 3 and 5 after STZ treatment (Supplementary [Supplementary-material supplementary-material-1]), and successful diabetes was confirmed by greater than 16.7 mM blood glucose level in both examinations.

Rats in the IDD, diabetes-induced IDD, and butein-treated diabetes-induced IDD groups were anaesthetized by intraperitoneal injection of 2% (*w*/*v*) pentobarbital (40 mg/kg), and 27-G needles were used to puncture the whole layer of AF though the tail skin. The needles were kept in the disc for 1 min. Butein was diluted with normal saline to achieve a final concentration of 10 mg/ml. After surgery, the butein solution was immediately intraperitoneally injected at 10 mg/kg every two days into the rats in the butein-treated diabetes-induced IDD group, and saline was injected into rats in the IDD and diabetes-induced IDD groups every two days until the rats were euthanized. The rats were daily monitored to ensure their well-being, and all animals were allowed free unrestricted weight bearing and activity.

### 2.3. NP Cell Culture

NP cells were extracted from healthy NP of young rats (20 males, 100–150 g), plated, and expanded for 3 weeks at 37°C and 5% CO_2_ in Dulbecco's modified Eagle's medium (DMEM) containing 20% foetal bovine serum (FBS; Gibco, Waltham, MA, USA) and 1% penicillin/streptomycin (Invitrogen). The culture medium was replaced twice every week, except in the case of primary cells, which were allowed more time (6.7 ± 1.4 days) to adhere prior to the first change of medium. Cells from the second passage were used in subsequent experiments. NP cell culture followed the method of the previous study [33].

### 2.4. Cell Culture and Treatment Protocols

In this study, we prepared high-glucose media with different concentrations. First, we checked the glucose concentration in the medium provided by Gibco Co. Then, we calculated the glucose required to be added to the medium with the expected concentration of high glucose. Finally, with aseptic operation, we put the glucose into the DMEM medium. We added different concentrations of glucose (5.5, 25, 50, 100, and 150 *μ*M) into the culture medium of NP cells for 24 h to establish the senescence and apoptosis models of NP cells. Cells were treated with different concentrations of butein (0, 2, 5, 10, and 20 *μ*M) and 50 *μ*M glucose (24 h) to investigate the effect of the treatment on apoptosis and senescence. To study the role of Sirt1 in cell protection, we pretreated NP cells with 10 mM Ex527 (a Sirt1 inhibitor) for 2 h before initiating butein treatment. All experiments were performed in triplicate.

### 2.5. Cell Viability Assay

According to our previous study [33], cell viability was assayed with the Cell Counting Kit-8 (CCK-8; Dojindo Co., Kumamoto, Japan) according to the manufacturer's protocol. As previously described [34], first, second-passage NP cells were plated in 96-well plates (8000 cells/cm^2^) and cultured in DMEM with 10% FBS at 37°C and 5% CO_2_ for 24 h. Then, the cells were treated with glucose at different concentrations for various durations. The toxicity of butein was also evaluated. Cells were treated with butein in a dose- and time-dependent manner. After treatment, the cells were washed with phosphate-buffered saline (PBS), and then, 100 *μ*l of DMEM containing 10 *μ*l of CCK-8 solution was added to each well, and the plate was incubated for an additional 2-4 h. The absorbance of the wells was then measured at 450 nm using a microplate reader.

### 2.6. Western Blot Assay

Due to our previous study [33], total protein of NP cells was isolated from cells using RIPA buffer with 1 mM phenylmethanesulfonyl fluoride (PMSF), and protein concentration was measured by using the BCA protein assay kit (Beyotime). Thirty micrograms of protein was separated by sodium dodecyl sulfate-polyacrylamide gel electrophoresis (SDS-PAGE) and transferred to polyvinylidene difluoride membranes (Bio-Rad, USA). Following blocking with 5% nonfat milk, the membranes were incubated with primary antibodies against cleaved caspase-3 (1 : 1000), Bax (1 : 1000), Bcl-2 (1 : 1000), p16INK4a (1 : 1000), acetyl-p53 (1 : 1000), p53 (1 : 1000), p21WAF1 (1 : 1000), *β*-actin (1 : 1000), and Sirt1 (1 : 500) overnight at 4°C, followed by incubation with the respective secondary antibodies. The bands were detected with an electrochemiluminescence plus reagent (Invitrogen). Last, the intensity of the protein bands was quantified with Image Lab 3.0 software (Bio-Rad).

### 2.7. Magnetic Resonance Imaging

After 4 or 12 weeks of puncture, the animals were examined via magnetic resonance imaging (MRI). All rats were anaesthetized by an intraperitoneal injection of 10% pentobarbital (40 mg/kg). The rats were fixed in a prone position for MRI, and then, the finger-specific coil MRI mode was used for rat tails as previously described [10]. Then, MRI was performed on all rats to evaluate the signal and structural changes in sagittal T2-weighted images using a 3.0 T clinical magnet (Philips Intera Achieva 3.0 MR). T2-weighted sections in the sagittal plane were obtained with the following settings: fast-spin echo sequence with time to repetition (TR) of 5400 ms and time to echo (TE) of 920 ms, 320 (h) × 256 (v) matrix, field of view of 260, and 4 excitations. The section thickness was 2 mm with a 0 mm gap. The images were evaluated by three double-blinded orthopaedic researchers who classified the extent of IDD according to the Pfirrmann grade (1 point = grade I, 2 points = grade II, 3 points = grade III, and 4 points = grade IV).

### 2.8. Histology and Immunohistochemistry

On weeks 4 and 12 after surgery, the rats were euthanized by an intraperitoneal overdosage of 4% pentobarbital, and the tails were harvested. As previously described [33], the specimens were decalcified and fixed in formaldehyde, dehydrated, and embedded in paraffin. The tissues were cut into 5 *μ*m sections. Slides of each disc were stained with Safranin O-fast green (S-O) and haematoxylin and eosin (H&E). The cellularity and morphology of NP and AF were examined by a separate group of experienced histology researchers in a blinded manner using a microscope (Olympus Inc., Tokyo, Japan) and evaluated using a grading scale. The histologic score was 5 for normal disc, 6–11 for moderately degenerated disc, and 12–14 for severely degenerated disc.

### 2.9. Immunofluorescence

As previously described [33], cells were mounted on coverslips, washed with PBS three times for 5 min each, fixed with 4% paraformaldehyde for 15 min, permeabilized with 0.5% (*v*/*v*) Triton X-100 for 20 min, and blocked with 1% (*w*/*v*) goat serum albumin for 1 h at 37°C. The samples were then probed at 4°C overnight with antibodies against SIRT1, acetyl-p53, or cleaved caspase-3 and incubated for 1 hour with a secondary antibody. Finally, nuclei were stained for 5 min with 0.1 g/ml 4′,6-diamidino-2-phenylindole (DAPI), and samples were washed and imaged with a fluorescence microscope. Protein expression was quantified by integrated optical density (IOD) with the Image-Pro Plus image analysis system.

### 2.10. TUNEL Assay

The terminal deoxynucleotidyl transferase (TdT) dUTP nick end labelling (TUNEL) assay is a technique for measuring apoptotic DNA fragmentations. As previously described [10, 33], after being fixed with a freshly prepared 4% paraformaldehyde for 1 h, NP cells were incubated with 3% H_2_O_2_ and 0.1% Triton X-100 for 10 min and washed three times with PBS between steps. According to the manufacturer's instructions, the cells were stained with the In Situ Cell Death Detection Kit (F. Hoffmann-La Roche Ltd., Basel, Switzerland) and DAPI. Finally, three random fields were imaged per slide with a fluorescence microscope (Olympus Inc., Tokyo, Japan).

### 2.11. Reactive Oxygen Species Assay

Cells were plated in six-well plates in complete culture medium. After sixteen hours, the cells (90% confluence) were washed with PBS. Then, the cells were incubated with reactive oxygen species (ROS) assay mixture according to the protocol of a reactive oxygen species (ROS) assay kit. Finally, three random microscopic fields were imaged per slide with a fluorescence microscope (Olympus Inc., Tokyo, Japan).

### 2.12. Sirt1 Activity Assay

Whole cell lysate was collected following treatment with RIPA buffer (Millipore, MA) supplemented with the protease inhibitor PMSF. Total protein concentration of the samples was measured using a commercially available BCA protein assay kit (Spectra Max190, Molecular Devices, USA). An enzymatic assay for SIRT1 activity was performed according to the manufacturer's instructions (Sigma-Aldrich). The plates were read with a fluorescent spectrophotometer (Spectra Max190, Molecular Devices) at 340 nm excitation and 430 nm emission.

### 2.13. Statistical Analysis

The results were presented as mean ± S.D. Statistical analyses were performed using the SPSS statistical software program 20.0 (IBM, Armonk, NY, USA). Data were analyzed by one-way analysis of variance (ANOVA) followed by Tukey's test for comparison between control and treatment groups. Nonparametric data (Pfirrmann grading) were analyzed by the Kruskal-Wallis *H* test. *p* < 0.05 was considered significant.

## 3. Results

### 3.1. Increased Apoptosis in Hyperglycaemic NP Cells

**Figure 1:**
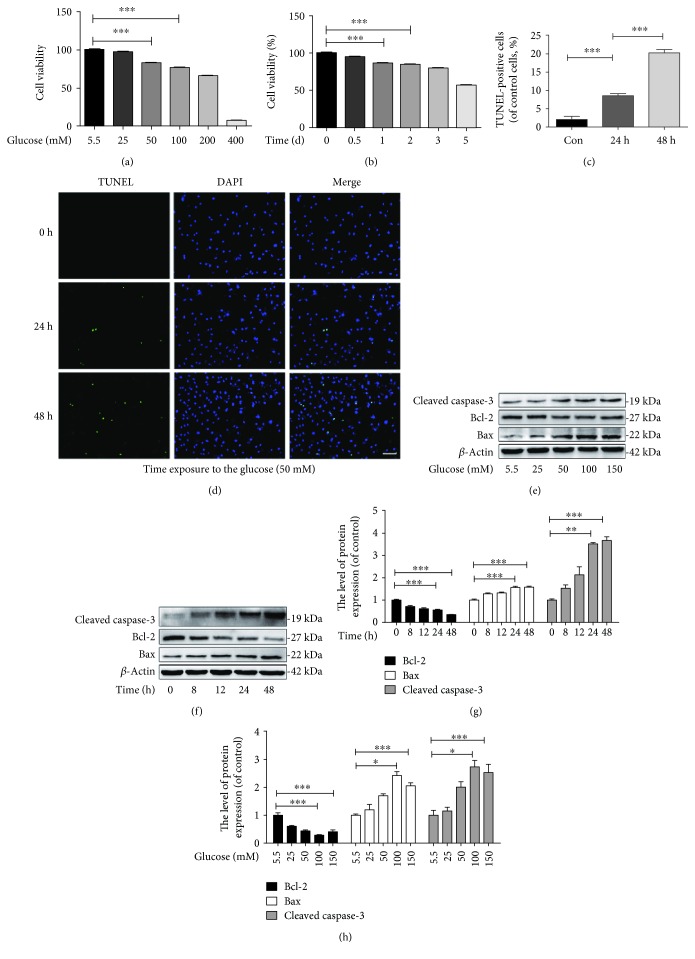
High glucose suppresses cell viability and promotes apoptosis in nucleus pulposus cells *in vitro*. (a) The cytotoxicity of glucose in NP cells was determined at different concentrations at a duration of 24 h using the CCK-8 assay. (b) The cytotoxicity of glucose in the nucleus pulposus was determined via the CCK-8 assay using a glucose concentration of 50 *μ*M for different durations. (c–d) The TUNEL assay was performed on the NP cells treated with different concentrations of glucose (original magnification ×200, scale bar: 50 *μ*m) and used in the quantification of apoptotic-positive cells. (e, g) The expression of cleaved caspase-3, Bax, and Bcl-2 evaluated by western blot in NP cells treated with glucose in a dose-dependent manner and a time-dependent manner. (f, h) The quantification of Bcl-2, Bax, and cleaved caspase-3 immunoblots. The experiment was repeated at least three times, with a representative example shown. The data in the figures represent the means ± S.D. Significant differences between groups are indicated as ^∗∗∗^*p* < 0.001, ^∗∗^*p* < 0.01, and ^∗^*p* < 0.05.

To investigate the effect of hyperglycaemia on NP cells *in vitro*, we treated NP cells with different concentrations of glucose for various durations. The cell viability assay results showed that cell viability was reduced by exposure to pathologically high glucose concentrations in a dose- and time-dependent manner, especially after treatment with more than 50 mM glucose and for treatment longer than 2 days (Figures 1(a) and 1(b)). TUNEL assay results showed that the incidence of apoptosis was obviously increased in NP cells treated with 50 mM glucose for 24 and 48 h (Figures 1(c) and 1(d)) (*p* < 0.01). The proapoptotic marker Bax and the antiapoptotic marker Bcl-2 are members of the BCL-2 family and the main regulators of the apoptotic pathway, and cleaved caspase-3 is the executor of the final step of apoptosis. According to the western blotting results, after treatment with glucose for longer than 24 h, the Bax/Bcl-2 ratio, one of the indexes of apoptosis, was notably decreased (*p* < 0.01), but the expression of Bax and cleaved caspase-3 was increased (*p* < 0.01). Additionally, with an increased concentration of glucose, the Bcl-2 expression was significantly downregulated, whereas the Bax/Bcl-2 ratio and the cleaved caspase-3 level were markedly elevated with increased treatment duration (Figures 1(e)–1(h)). Therefore, our results suggest that treatment with different concentrations of glucose for different durations can markedly promote apoptosis in NP cells.

### 3.2. Increased Senescence in Hyperglycaemic NP Cells

**Figure 2:**
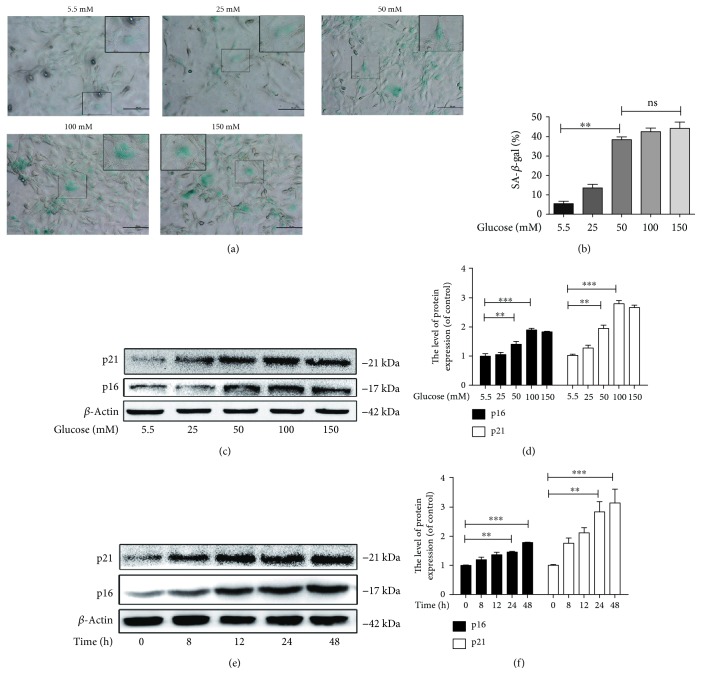
High glucose promotes senescence in nucleus pulposus cells *in vitro*. (a, b) The SA-*β*-gal staining results of NP cells treated with different concentrations of glucose for 24 h (original magnification ×200, scale bar: 50 *μ*m) and the quantification of senescence cells are shown. (c, e) The protein expression of the senescence markers, p21WAF1 and p16INK4a, was evaluated by western blot in NP cells treated with glucose in a dose-dependent manner and a time-dependent manner. (d, f) The quantification of p21WAF1and p16INK4a immunoblots is shown. The experiment was repeated at least three times, with a representative example shown. The data in the figures represent the means ± S.D. Significant differences between groups are indicated as ^∗∗∗^*p* < 0.001, ^∗∗^*p* < 0.01, and ^∗^*p* < 0.05.

As shown in Figure 2(a), senescence in NP cells was measured by SA-*β*-gal staining and found to be markedly increased after glucose treatment in a dose-dependent manner (Figures 2(a) and 2(b)). The western blot results showed that high concentrations of glucose can markedly promote the expression of p16INK4a and p21WAF1, classical senescence-related proteins, in a dose- and time-dependent manner (Figures 2(c)–2(f)) (*p* < 0.01). Therefore, our results suggest that high concentrations of glucose can promote senescence in NP cells *in vitro*.

### 3.3. Decreased Expression and Activity of Sirt1 as well as Increased Acetylation of p53 in Diabetic NP Tissues and Hyperglycaemic NP Cells

**Figure 3:**
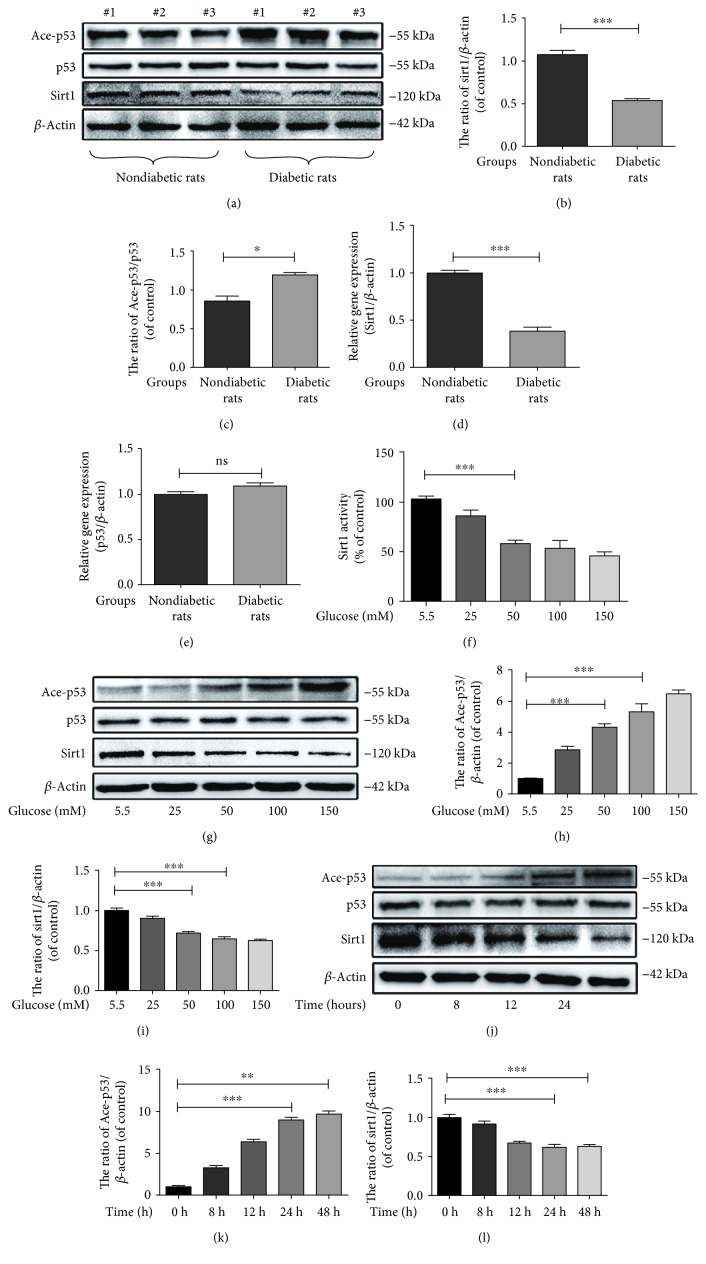
Increased expression of acetyl-p53 and decreased expression of Sirt1 in diabetic NP tissues and high glucose-stimulated NP cells. (a) The expression of acety-p53 and Sirt1 was evaluated by western blotting in NP cells derived from nondiabetic rats and diabetic rats. (b, c) The quantification of acety-p53 and Sirt1 immunoblots. (d, e) The mRNA expression of Sirt1 and p53 was evaluated by western blotting in NP cells derived from nondiabetic rats and diabetic rats. (f) The activity of Sirt1, determined using the Sirt1 assay kit, in NP cells treated with various concentrations of glucose. (g, j) The expression of acetyl-p53, p53, and Sirt1 was evaluated by western blotting in NP cells treated with glucose in a dose-dependent manner and a time-dependent manner. (h, i, k, l) The quantification of immunoblots of acetyl-p53/p53 and Sirt1 is shown. The experiment was repeated at least three times, with a representative example shown. The data in the figures represent the means ± S.D. Significant differences between groups are indicated as ^∗∗∗^*p* < 0.001, ^∗∗^*p* < 0.01, and ^∗^*p* < 0.05.

As shown in Figure 3(a), western blot results indicate that the expression of Sirt1 in NP was lower in NP tissues of diabetic rats than in those of nondiabetic rats (Figures 3(c) and 3(d)) (*p* < 0.01). In addition, Sirt1 expression was reduced not only *in vivo* but also *in vitro* in response to high glucose concentrations (Figures 3(a), 3(c), 3(e), and 3(h)) (*p* < 0.01). The activity of Sirt1 was measured using the Sirt1 activity assay, and the results showed that Sirt1 activity was decreased in NP cells treated with glucose in a dose-dependent manner (Figure 3(d)).

Previous studies have demonstrated that hyperglycaemia reduces SIRT1 expression, leading to increased p53 acetylation [35]. Acetyl-p53 is tightly associated with senescence and apoptosis. Therefore, we measured the effects of hyperglycaemia on the acetylation of p53 (in diabetic NP tissues and hyperglycaemic NP cells) in NP cells. In addition, the western blot showed that acetyl-p53 was obviously increased in diabetic NP tissues compared to nondiabetic NP tissues (Figures 3(a) and 3(b)) (*p* < 0.01). Western blotting showed that the acetyl-p53/p53 ratio, a marker associated with apoptosis and senescence, was increased in response to glucose in a dose- and time-dependent manner (Figures 3(e), 3(f), 3(h), and 3(i)) (*p* < 0.01). These results indicate that the expression and activity of Sirt1 were decreased, and the acetylation of p53 was increased in diabetic NP tissues and hyperglycaemic NP cells.

### 3.4. The Activation of Sirt1 by Butein Promotes Viability in Hyperglycaemic NP Cells and Suppresses p53 Acetylation

**Figure 4:**
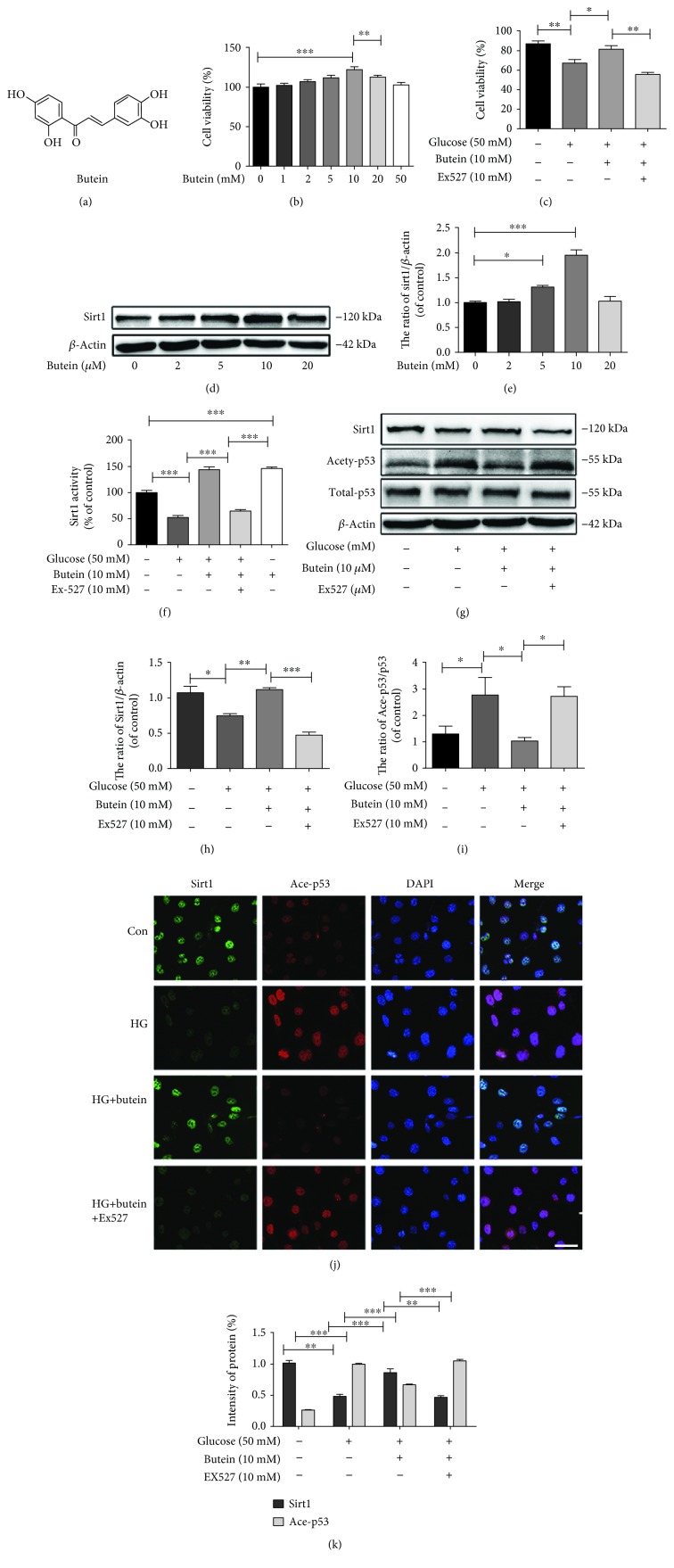
The activation of Sirt1 by butein promotes viability in hyperglycaemic NP cells and suppresses p53 acetylation. (a) Chemical structural formula of butein. (b) Cell viability of NP cells when treated with butein in a dose-dependent manner. (c) Cell viability of NP cells in four groups. (d) The expression of Sirt1 was evaluated by western blot in NP cells treated with butein in a dose-dependent manner. (e) The quantification of Sirt1 immunoblots. (f) The activity of Sirt1 in NP cells in different groups using the Sirt1 assay kit. (g) The expression of ace-p53, p53, and Sirt1 was determined by western blot in NP cells. (h, i) The quantification of Sirt1, ace-p53, and p53 immunoblots. (j) Double labelling immunofluorescence staining of Sirt1 and ace-p53 results in NP cells as treated above and observed in an Olympus fluorescence microscope (original magnification ×400, scale bar: 25 *μ*m). (k) More than three randomly selected images and the quantification of the immunofluorescent intensity of Sirt1 and ace-p53 are shown. The experiment was repeated at least three times independently, with a representative example shown. The data in the figures represent the means ± S.D. Significant differences between groups are indicated as ^∗∗∗^*p* < 0.001, ^∗∗^*p* < 0.01, and ^∗^*p* < 0.05.

Butein, an anticancer drug, has been reported to activate the expression and activity of Sirt1 and was employed in this study to investigate the relationship between Sirt1 and diabetic IDD. To confirm the effects of butein in increasing the expression and activity of Sirt1 in NP cells, we performed western blotting and a Sirt1 activity assay, and the results showed that both the expression and activity of Sirt1 were increased, especially with 10 *μ*M butein (Figures 4(d)–4(f)) (*p* < 0.01). To confirm the relationship between Sirt1 and acetyl-p53, we employed the inhibitor of Sirt1 Ex527. The western blotting results showed that the expression of Sirt1 decreased and the acetyl-p53/p53 ratio was increased in response to Ex527. In contrast, in response to butein treatment, the expression and activity of Sirt1 were increased, and the acetyl-p53/p53 ratio was decreased. After Ex527 was added to the NP cells, the effects of butein were reversed (Figures 4(d)–4(f)) (*p* < 0.01). Double immunofluorescence staining for Sirt1 and acetyl-p53 also confirmed the above results (Figures 4(j) and 4(k)). Therefore, the regulation of Sirt1 by butein and Ex527 can modulate the acetyl-p53 ratio in NP cells in response to high glucose concentrations *in vitro*.

### 3.5. The Activation of Sirt1 by Butein Ameliorates High Glucose-Induced Apoptosis in NP Cells

**Figure 5:**
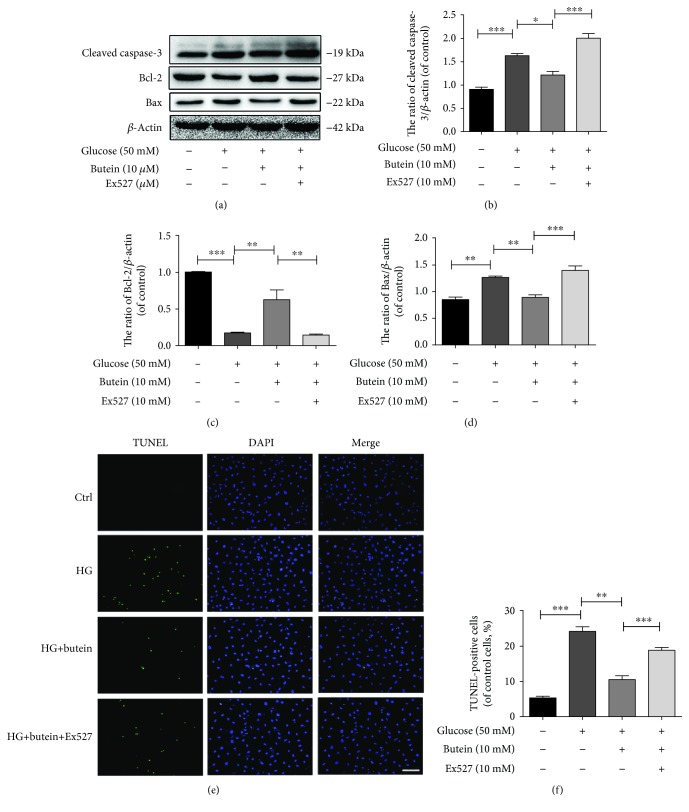
Activation of Sirt1 suppresses apoptosis in nucleus pulposus cells. Nucleus pulposus cells were treated with DMEM (20% FBS), glucose (50 mM), glucose (50 mM) and butein (10 *μ*M), or glucose (50 mM) and butein (10 *μ*M) plus Ex527 (10 *μ*M). (a) The protein expression of cleaved caspase-3, Bax, and Bcl-2 was evaluated by western blot in NP cells as treated above. (b–d) The quantification of Bcl-2, Bax, and cleaved caspase-3 immunoblots. (e, f) The TUNEL assay was performed in NP cells (original magnification ×200, scale bar: 50 *μ*m), and the quantification of apoptotic-positive cells is shown. The experiment was repeated three times independently, with a representative example shown. The data in the figures represent the means ± S.D. Significant differences between groups are indicated as ^∗∗∗^*p* < 0.001, ^∗∗^*p* < 0.01, and ^∗^*p* < 0.05.

To further investigate how activation of Sirt1 by butein may affect NP cells, we examined apoptosis in NP cells under conditions of hyperglycaemia. The western blotting results showed that the levels of apoptosis markers, such as cleaved caspase-3 and Bax, were elevated in NP cells stimulated by high glucose and that butein treatment decreased the levels of cleaved caspase-3 and Bax. However, in the presence of the Sirt1 selective inhibitor Ex527, butein could no longer downregulate cleaved caspase-3 and Bax in hyperglycaemic NP cells (Figures 5(a), 5(b), and 5(d)) (*p* < 0.01). Moreover, hyperglycaemia reduced the expression of Bcl-2, and butein promoted Bcl-2 expression in hyperglycaemic NP cells; however, when Sirt1 was inhibited by Ex527, butein could no longer upregulate Bcl-2 expression in hyperglycaemic NP cells. Furthermore, we assessed apoptosis using the TUNEL assay and found that the incidence of apoptosis was increased in cells treated with high glucose and that butein reduced the incidence of apoptosis in NP cells treated with high glucose; however, in the presence of Ex527, butein did not reduce apoptosis in the hyperglycaemic NP cells (Figure 5(e)) (*p* < 0.01). Therefore, our results suggest that the activation of Sirt1 by butein can ameliorate high glucose-induced apoptosis in NP cells.

### 3.6. The Activation of Sirt1 by Butein Reduces High Glucose-Induced Senescence in NP Cells

**Figure 6:**
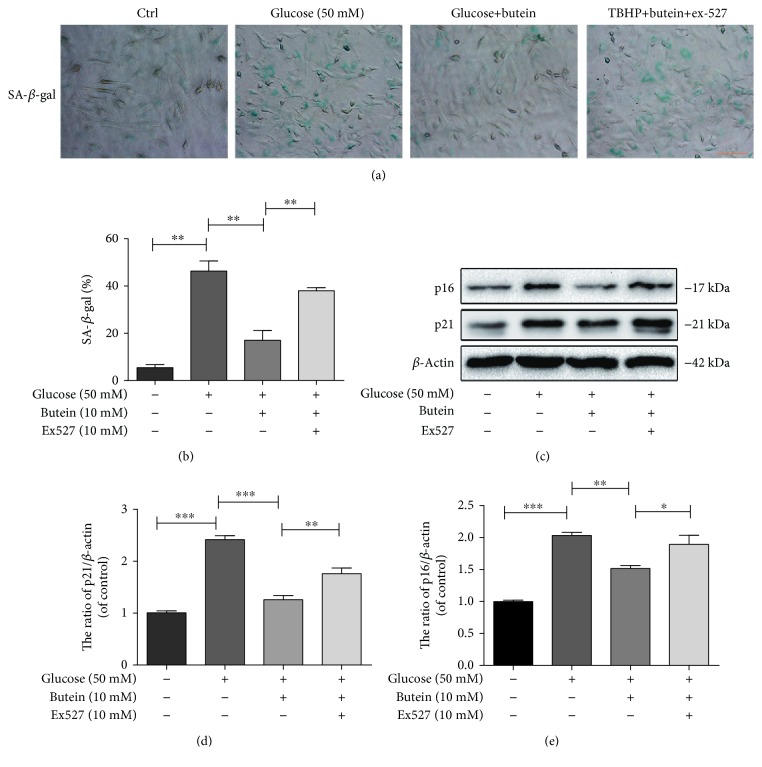
The activation of Sirt1 suppresses senescence in nucleus pulposus cells. NP cells were treated with DMEM (20% FBS), glucose (50 mM), glucose (50 mM) and butein (10 *μ*M), or glucose (50 mM) and butein (10 *μ*M) plus Ex-527 (10 *μ*M). (a, b) The SA-*β*-gal staining results of nucleus pulposus cells as treated above are shown (original magnification ×200, scale bar: 50 *μ*m), and the quantification of senescence cells is shown. (c) The expression of senescence markers, such as p16INK4a and p21WAF1, was evaluated by western blot in NP cells as treated above. (d, e) The quantification of p16INK4a and p21WAF1 immunoblots is shown. The experiment was repeated three times independently, with a representative example shown. The data in the figures represent the means ± S.D. Significant differences between groups are indicated as ^∗∗∗^*p* < 0.001, ^∗∗^*p* < 0.01, and ^∗^*p* < 0.05.

As shown in Figure 6(a), the proportion of SA-*β*-gal-positive senescent NP cells was increased in response to high glucose, while the proportion of SA-*β*-gal-positive senescent NP cells was reduced in the butein treatment group. However, when Sirt1 was inhibited by Ex527, butein could not reduce senescence in NP cells (Figures 6(a) and 6(b)) (*p* < 0.01). We also evaluated senescence-related proteins using western blotting. The western blotting results showed that the expression of senescence-related proteins, including p21WAF1 and p16INK4a, was markedly increased in the high-glucose group (Figures 6(c)–6(e)) (*p* < 0.01) and decreased in the butein-treated group and that the effect of butein was diminished when Sirt1 was inhibited by Ex527 (Figures 6(c)–6(e)) (*p* < 0.01). These results suggest that the activation of Sirt1 by butein may reduce high glucose-induced NP cell senescence.

### 3.7. Butein Ameliorates Diabetic IDD in Rats *In Vivo*

**Figure 7:**
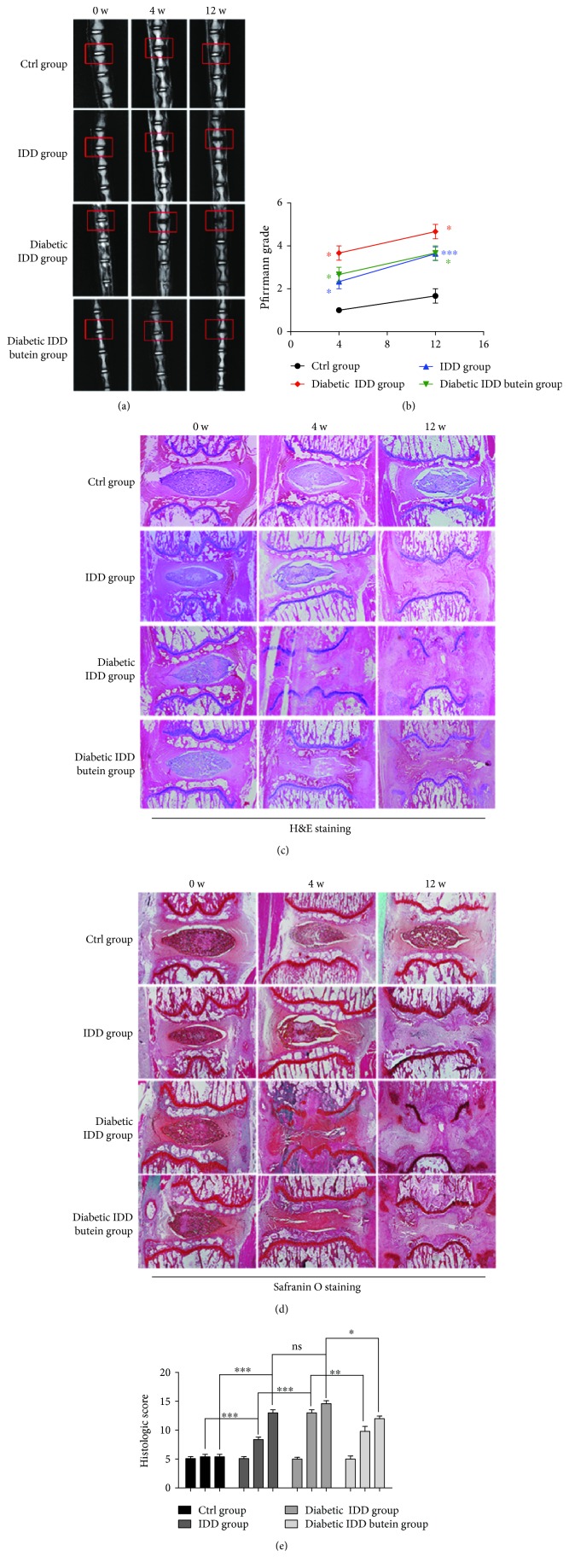
The activation of Sirt1 ameliorates IDD in rats *in vivo*. The diabetic rat IDD model was established by stabbing the whole layer of the annulus fibrosus (AF) of diabetic rats, which was induced by an STZ (60 mg/kg) intraperitoneal injection, through the tail skin using needles (27 G) for 1 min. MRI of 0-, 4-, and 12-week degenerated discs was performed followed by staining with H&E and Safranin O. (a, b) T2-weighted MRI of a rat tail with a needle-punctured disc at 4 and 12 weeks postsurgery (red boxes: location of the needle-puncture disc). The Pfirrmann MRI grade scores in three groups at week 4 and week 12 postsurgery are shown (6 rats at each time point for each group). (c, d) Representative S-O and HE staining of disc samples from different experimental groups at 4 weeks and 12 weeks postsurgery is shown (original magnification ×40, scale bar: 100 *μ*m). Three sections were randomly selected for quantification, with a representative example shown. (e) The histological grades evaluated at 4 weeks and 12 weeks postsurgery in three groups (6 rats per group) are shown. The displayed values are the means ± S.D. of 6 rats per group. Significant differences between groups are indicated as ^∗∗∗^*p* < 0.001, ^∗∗^*p* < 0.01, and ^∗^*p* < 0.05. Blue: IDD group vs. Ctrl group; red: diabetic IDD group vs. IDD group; green: diabetic IDD butein group vs. diabetic IDD group.

We established a puncture-induced IDD model in diabetic rats to evaluate the therapeutic effects of butein on IDD *in vivo*. The severity of IDD in rats was assessed using MRI and quantified according to the Pfirrmann MRI grading system. The MR images obtained at 4 weeks after puncture showed that the T2-weighted signal intensity was reduced in the IDD group compared to the sham-operated non-IDD group (control group). The signal intensity was weaker in the diabetic IDD group, while butein was shown to increase the T2-weighted signal intensity in the diabetic IDD group. The same trend was observed at the 12-week time point, although the intensities of each group were weaker than those at the 4-week time point (Figure 7(a)). The phenomenon was confirmed by the quantitative evaluation using the Pfirrmann MRI grading system (Figure 7(b)).

We also evaluated the process of IDD by histology. S-O staining (used for staining proteoglycans and glycosaminoglycans) and H&E staining (used for observation of the general histological structure of NP tissues) were performed to evaluate the effect of diabetes and butein treatment on IDD. Based on H&E staining, it was observed that the structure of the NP begins to become disordered in the IDD group compared to the sham group (control group) at 4 weeks. It also indicated that the structure had almost disappeared in the diabetic IDD group at 4 weeks compared to the IDD group. However, the NP structure in the butein-treated group was better preserved than that in the diabetic IDD group at 4 weeks (Figure 7(c)). According to the S-O staining, the extracellular matrix of the NP tissues was destroyed in the IDD group compared with the sham group (control group), and the destruction of the extracellular matrix in the diabetic IDD group was notably higher than that of the simple puncture-induced IDD model (IDD group). In addition, not only the structure of the NP but also the extracellular matrix of the NP tissue was better preserved in the butein-treated group than in the diabetic IDD group at the 4-week time point (Figure 7(d)). Similar H&E and S-O staining results were observed at the 12- week time point (Figures 7(c) and 7(d)). We also quantified the process of IDD in each group according to histologic scores, which showed that diabetes aggravated the process of IDD at the 4-week and 12-week time points, while treatment with butein alleviated IDD at the 4-week and 12-week time points (Figure 7(e)) (*p* < 0.01). These results suggested that diabetes aggravates the process of IDD, and butein can ameliorate diabetic IDD in rats.

**Figure 8:**
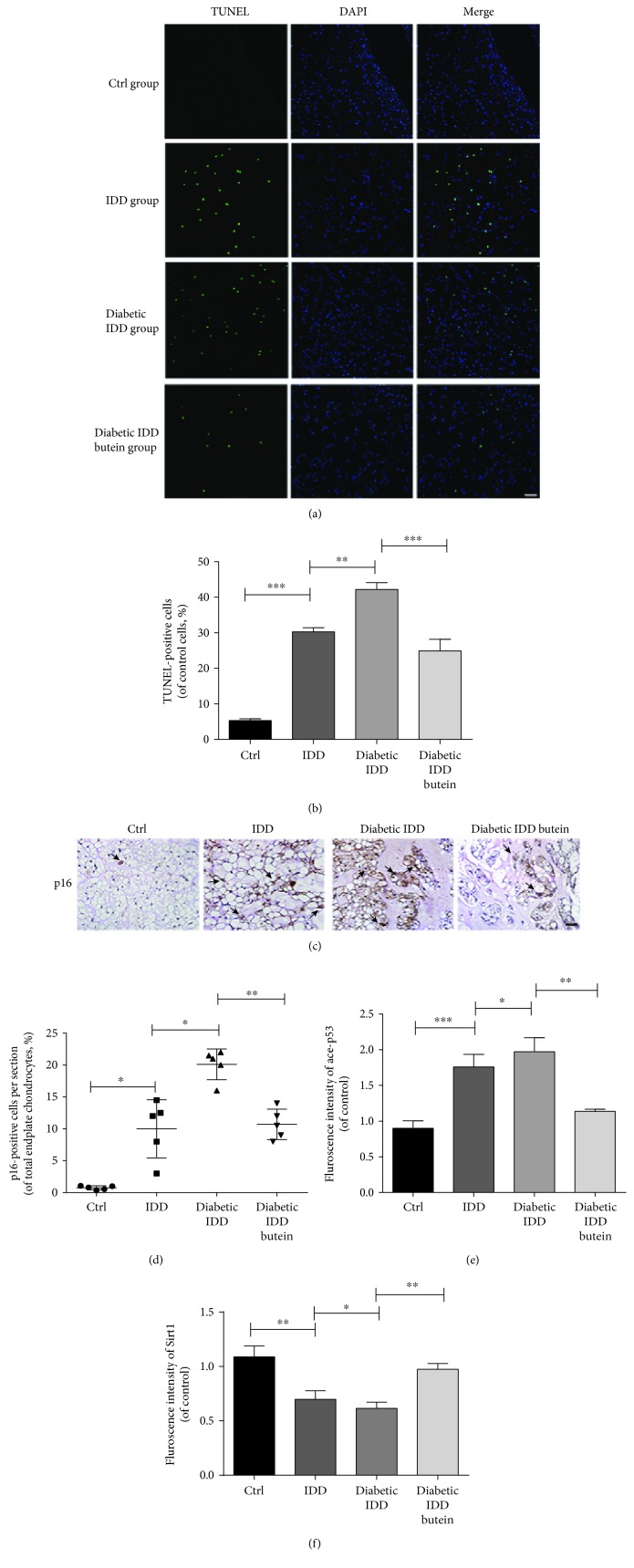
The activation of Sirt1 suppresses p53 acetylation and decreases apoptosis and senescence in the nucleus pulposus *in vivo*. The diabetic rat IDD model was established by stabbing the whole layer of the annulus fibrosus (AF) of diabetic rats through the tail skin using 27-G needles for 1 min. The TUNEL assay was performed on 12-week degenerated discs, and Sirt1 and ace-p53 were obtained via immunofluorescence staining. 48 SD rats were randomly classified into four groups including the control group, IDD group, diabetes IDD group, and diabetes IDD butein group. (a) The representative TUNEL assay of disc samples from different experimental groups at 12 weeks postsurgery (original magnification ×200, scale bar: 50 *μ*m). (b) Three sections were randomly selected for quantification, with a representative example shown. (c) The immunohistochemical staining of the senescence typical marker p16INK4a in intervertebral disc sections at 4 weeks postsurgery (the black arrow points to the positive staining cells). (d) The quantification of immunohistochemical staining of p16INK4a in disc samples from different experimental groups at 4 weeks postsurgery (6 rats per group) is shown (original magnification ×400, scale bar: 25 *μ*m). (e, f) The quantification of immunofluorescence label staining of Sirt1 and ace-p53 in disc samples from different experimental groups at 4 weeks postsurgery (6 rats per group) is shown. The displayed values are the means ± S.D. of 6 rats per group. Significant differences between groups are indicated as ^∗∗∗^*p* < 0.001, ^∗∗^*p* < 0.01, and ^∗^*p* < 0.05.

Furthermore, we assessed apoptosis and senescence in each group *in vivo*. TUNEL assay results of NP tissue sections showed that the proportion of the apoptotic cells in the IDD group was higher than that in the sham group (control group); the number of apoptotic cells in the diabetic IDD group was higher than that in the IDD group (Figures 8(a) and 8(b)) (*p* < 0.01). However, the proportion of apoptotic cells was lower in the butein-treated diabetic IDD group (Figures 8(a) and 8(b)) (*p* < 0.01). P16 was recognized as one of the authoritative indicators of NP cells and tissue senescence. The results of immunohistochemical staining of P16 indicated a greater proportion of positive cells in the NP tissue sections of the IDD group than in the sham group (control group); the number of p16-positive cells in the diabetic IDD group was higher than that in the IDD group (Figures 8(c) and 8(d)). However, the proportion of p16-positive cells was lower in the butein-treated diabetic IDD group (Figures 8(c) and 8(d)) (*p* < 0.01). Diabetes promotes apoptosis and senescence in degenerated NP tissue, and butein treatment can suppress apoptosis and senescence in the NP tissues of diabetic IDD rats.

**Figure 9:**
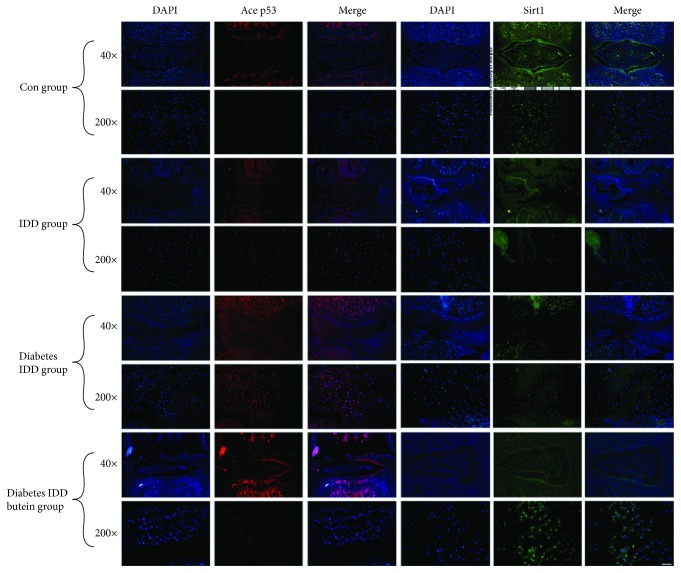
The immunofluorescence of Sirt1 and ace-p53 *in vivo*. (a) Representative immunofluorescence label staining of Sirt1 and ace-p53 in disc samples from different experimental groups at 4 weeks postsurgery is shown (original magnification ×200, scale bar: 50 *μ*m).

Immunofluorescence staining was employed to evaluate the expression of Sirt1 and p53 in NP tissues *in vivo*. As shown in Figures 8(e), 8(f), and 9(a), the fluorescence intensity of acetyl-p53 was increased in the IDD group at the 4-week time point, and the fluorescence intensity of acetyl-p53 was increased in the diabetic IDD group compared with the IDD group. However, the fluorescence intensity of acetyl-p53 was noticeably decreased in the butein-treated group at 4 weeks postsurgery compared to the diabetic IDD group (*p* < 0.01). Moreover, the fluorescence intensity of Sirt1, which is upstream of Ace-p53, was decreased in the IDD group at the 4-week time point, and the fluorescence intensity of Sirt1 was decreased in the diabetic IDD group compared with the IDD group. However, the fluorescence intensity of Sirt1 was noticeably upregulated in the butein-treated group at 4 weeks postsurgery compared to the diabetic IDD group (*p* < 0.01). In conclusion, the fluorescence intensity of Sirt1 showed the opposite trend to that of acetyl-p53 in each group at 4 weeks postsurgery.

Therefore, the results of the *in vivo* experiments showed that diabetes may promote the process of IDD by accelerating the apoptosis and senescence of NP cells *in vivo*; however, butein can ameliorate diabetic IDD by protecting NP cells against apoptosis and senescence via the Sirt1/P53 axis.

## 4. Discussion

The main findings of this study are that the acetylation of p53 was upregulated and the expression and activity of Sirt1 were downregulated in response to glucose in NP cells as well as in diabetic NP tissues. We also found that the upregulation of Sirt1 expression and activity reversed the acetylation of p53, which in turn suppressed apoptosis and senescence induced by high glucose in NP cells. *In vivo* experiments demonstrated that Sirt1 plays a protective role against diabetic IDD in a rodent model. Overall, this study showed that the Sirt1/p53 axis may play an essential role in the pathogenesis of diabetic IDD and might serve as a target in diabetic IDD.

Clinical studies have shown that diabetes is one of the risk factors for IDD and other disc diseases [36, 37]. Although Sakellaridis reported that IDD is associated with diabetes [14], the mechanism remains unclear. Several *in vivo* studies including our study have verified that the incidence of apoptosis and senescence is increased in rats with diabetes [15, 38]. Moreover, *in vitro* studies have shown that hyperglycaemia, the main characteristic of diabetes, may induce apoptosis and senescence in rat NP tissues [15, 39, 40]. In this study, we demonstrated that high glucose may promote apoptosis and senescence in a dose- and time-dependent manner (Figures 1 and 2), which is consistent with the reported results. However, the mechanism underlying high glucose-induced apoptosis and senescence in NP cells is still unknown.

p21WAF1, Bax, and Bcl-2 have been reported as targets of acetyl-53 [41], and the inhibition of p53 acetylation could reverse cell apoptosis and senescence [42, 43], showing that acetyl-p53 is tightly associated with apoptosis and senescence. To investigate the relationship between acetyl-p53 and diabetic IDD, we examined the acetyl-p53 expression *in vivo* as well as *in vitro*. We collected diabetic rat NP tissues and evaluated the acetyl-p53 expression in these tissues. Western blot results showed that the acetyl-p53/p53 ratio was dramatically increased in diabetic NP tissues compared to nondiabetic NP tissues (Figure 3(a)), which was further confirmed by immunofluorescence staining (Figure 9(a)). Using various concentrations of glucose for different treatment durations, we determined that high glucose can increase the acetyl-p53/p53 ratio in a dose- and time-dependent manner (Figures 3(g) and 3(j)). Both *in vivo* and *in vitro* studies suggested that p53 acetylation was induced by diabetes; however, the mechanism by which hyperglycaemia regulates the acetylation of p53 remains unknown. We hypothesized that Sirt1, which is upstream of acetyl-p53, is involved in this process.

Sirt1 is an NAD^+^-dependent deacetylase with a wide range of substrates, such as p53, FOXO, NF-*κ*B, and PGC-1*α*. It is considered a key modulator of acetyl-p53 deacetylation [44]. Previous studies have demonstrated that Sirt1 is one of the vital targets of glucose [45]. The results of the present study showed that the expression and activity of Sirt1 were downregulated in diabetic NP tissues as well as in high glucose-treated NP cells. Further studies involving Sirt1 activation and inhibition confirmed that Sirt1 is responsible for p53 acetylation as well as the apoptosis and senescence of NP cells. Additionally, *in vitro* and *in vivo* studies proved that the activation of Sirt1 by butein may provide a therapeutic potential for diabetic IDD. Thus, these studies demonstrated that the Sirt1/P53 axis may play an essential role in diabetic intervertebral disc degeneration pathogenesis and therapeutics.

In addition to Sirt1, we speculate that high glucose may regulate p53 acetylation through another mechanism. Glucose can be broken down into pyruvate through glycolysis, while pyruvate can be converted into lactate in the absence of oxygen. In the presence of oxygen, pyruvate will be converted to acetyl-CoA, which will then enter the tricarboxylic acid (TCA) cycle to produce ATP. It has been reported that the TCA flux is reduced and acetyl-CoA is accumulated in diabetes mellitus [46]. The accumulation of acetyl-CoA may in turn activate acetyltransferases such as CREB-binding protein (CBP), an acetyl-CoA-dependent enzyme, to acetylate p53 [47]. This finding may explain the partial rescue effect of Sirt1 activation on p53 deacetylation and suggests that we should suppress the production of acetyl-CoA when targeting p53 deacetylation, except in Sirt1 activation.

In this study, we highlight that diabetes induces apoptosis and senescence in NP cells, and IDD was age-related diseases but the common mechanism may exist in other age-related diseases. The previous study showed that inhibition of Sirt1 reduced deacetylation and promoted acetylation of p53, leading to apoptosis in rodent models of systemic inflammation, Parkinson's disease, or aging-related muscular atrophy [48].

**Figure 10:**
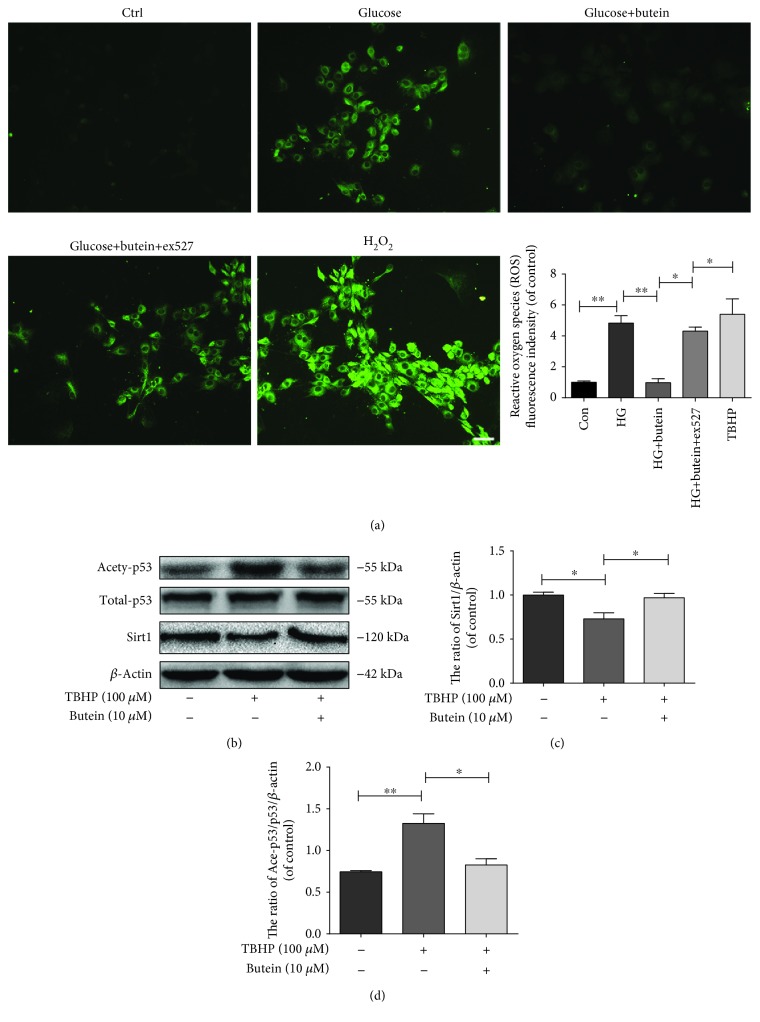
Butein treatment eliminates ROS in nucleus pulposus cells via the Sirt1 pathway. (a) ROS were measured in NP cells using an ROS assay kit (original magnification ×400, scale bar: 25 *μ*m). (b) The expression of Sirt1, ace-p53, and p53 was evaluated in NP cells by western blot, and representative results of the western blots are presented. (c, d) The quantification of Sirt1, ace-p53, and p53 immunoblots is shown. The experiment was repeated three times independently, with a representative example shown. The data in the figures represent the means ± S.D. Significant differences between groups are indicated as ^∗∗∗^*p* < 0.001, ^∗∗^*p* < 0.01, and ^∗^*p* < 0.05.

Moreover, accumulating evidence suggests that hyperglycaemia may induce oxidative stress, which is a major pathogenic factor in diabetes-associated complications [49]. Additionally, oxidative stress has been demonstrated as one of the main causes of apoptosis and senescence in NP cells in our studies [10, 39, 40]. Here, we report that hyperglycaemia promoted the production of ROS and that the activation of Sirt1 by butein may suppress ROS in NP cells (Figure 10(a)). Moreover, ROS induced by tert-butyl hydroperoxide (TBHP) may result in p53 acetylation through inhibiting the expression of Sirt1 in NP cells due to the western blot result (Figures 10(b), 10(c), and 10(d)). These results implied that Sirt1 may suppress p53 acetylation indirectly through ROS production regulation.

## 5. Conclusion

In conclusion, our study provides evidence that hyperglycaemia may enhance apoptosis and senescence in NP cells both *in vivo* and *in vitro* via the Sirt1/acetyl-p53 axis. The activation of Sirt1 may reduce p53 acetylation and potentially protect NP cells against apoptosis and senescence. These findings suggest that the Sirt1/P53 axis may play an essential role in diabetic intervertebral disc degeneration pathogenesis and therapeutics.

## Data Availability

The data used to support the findings of this study are included within the article.

## Supplementary Materials

Supplementary Materials Supplementary Figure 1: rats' blood glucose level. (a) Blood glucose level in different groups was examined on days 3 and 5 after STZ treatment or saline.
