# Supplementary figures and images for: The Sirt1/P53 Axis in Diabetic Intervertebral Disc Degeneration Pathogenesis and Therapeutics
Source: Oxid Med Cell Longev. 2019 Sep 9;2019:7959573. doi: 10.1155/2019/7959573 (PMC6754956; doi:10.1155/2019/7959573)

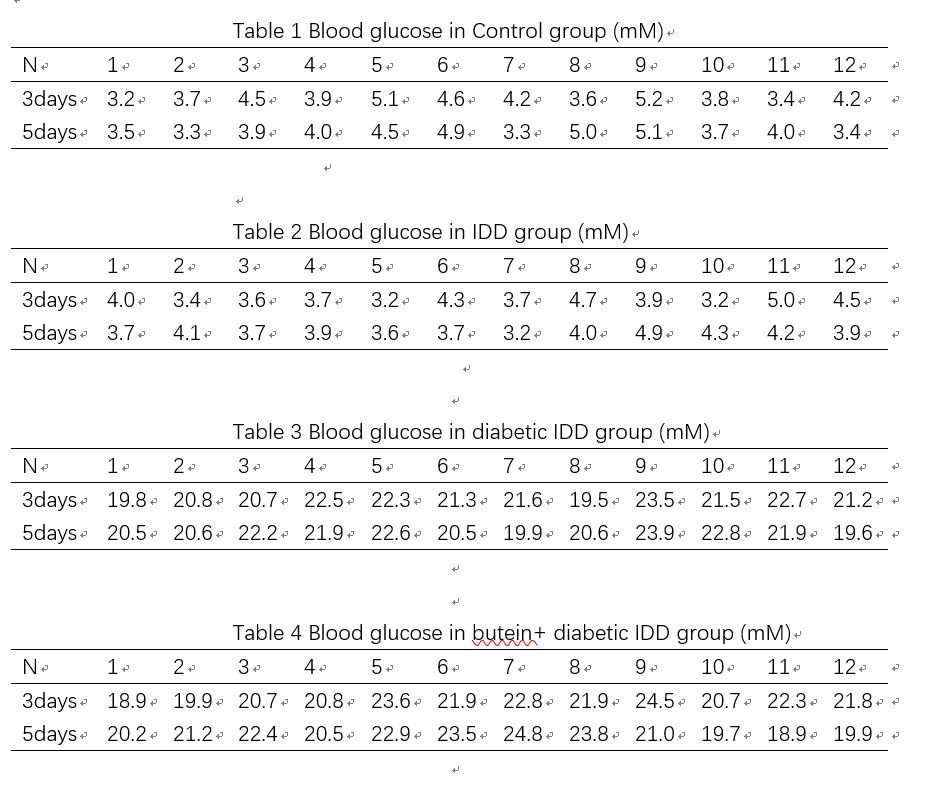

Supplement: Supplementary Materials — Supplementary Figure 1: rats' blood glucose level. (a) Blood glucose level in different groups was examined on days 3 and 5 after STZ treatment or saline. [file 7959573.f1.JPG]
